# Automatic Classification of Squat Posture Using Inertial Sensors: Deep Learning Approach

**DOI:** 10.3390/s20020361

**Published:** 2020-01-08

**Authors:** Jaehyun Lee, Hyosung Joo, Junglyeon Lee, Youngjoon Chee

**Affiliations:** 1 Interdisciplinary Program of Medical and Biological Engineering, University of Ulsan, 93, Daehak-ro, Nam-gu, Ulsan 44610, Korea; jhe0308@gmail.com (J.L.); mywngprud1@gmail.com (H.J.); 2 Department of Exercise and Sport Science, University of Ulsan, 93, Daehak-ro, Nam-gu, Ulsan 44610, Korea; erovic@naver.com; 3 School of Electrical Engineering, University of Ulsan, 93, Daehak-ro, Nam-gu, Ulsan 44610, Korea

**Keywords:** deep learning, inertial measurement unit, exercise classification, squat, self-fitness

## Abstract

Without expert coaching, inexperienced exercisers performing core exercises, such as squats, are subject to an increased risk of spinal or knee injuries. Although it is theoretically possible to measure the kinematics of body segments and classify exercise forms with wearable sensors and algorithms, the current implementations are not sufficiently accurate. In this study, the squat posture classification performance of deep learning was compared to that of conventional machine learning. Additionally, the location for the optimal placement of sensors was determined. Accelerometer and gyroscope data were collected from 39 healthy participants using five inertial measurement units (IMUs) attached to the left thigh, right thigh, left calf, right calf, and lumbar region. Each participant performed six repetitions of an acceptable squat and five incorrect forms of squats that are typically observed in inexperienced exercisers. The accuracies of squat posture classification obtained using conventional machine learning and deep learning were compared. Each result was obtained using one IMU or a combination of two or five IMUs. When employing five IMUs, the accuracy of squat posture classification using conventional machine learning was 75.4%, whereas the accuracy using deep learning was 91.7%. When employing two IMUs, the highest accuracy (88.7%) was obtained using deep learning for a combination of IMUs on the right thigh and right calf. The single IMU yielded the best results on the right thigh, with an accuracy of 58.7% for conventional machine learning and 80.9% for deep learning. Overall, the results obtained using deep learning were superior to those obtained using conventional machine learning for both single and multiple IMUs. With regard to the convenience of use in self-fitness, the most feasible strategy was to utilize a single IMU on the right thigh.

## 1. Introduction

The squat is a fitness exercise performed by both athletes and non-athletes to reduce pain, maintain muscle status, and improve the quality of exercise performance [1,2,3]. However, when inexperienced individuals perform squats without professional coaching, the risk of spinal and/or knee injuries increases [4]. Office workers and other non-athletes may struggle to spend sufficient time and money to visit a fitness center regularly and receive professional coaching. Thus, the development of a self-coaching system could help individuals evaluate their own exercise performance without professional assistance.

Recent studies in the literature have employed inertial measurement units (IMUs) and 3-D motion capture systems to recognize and assess human motion during exercise [5]. However, 3-D motion capture systems are unsuitable for personal fitness because they require large, complex, and expensive measurement environments comprising multiple motion tracking cameras and markers affixed to the bodies of subjects. The other method is an image-processing approach that employs deep convolutional neural networks to learn the image features for activity recognition [6,7] and human pose estimation [8,9,10]. Because the video image processing needs high computation power, it is not proper for self-coaching system in home. By contrast, current IMUs support the acquisition of data in nine axes by using accelerometers, gyroscopes, and magnetic trackers, and they can be used to measure the motion and kinematics of body segments. Such IMU systems facilitate the construction of comfortable, compact, and relatively inexpensive measurement environments. However, along with an IMU system, a proper algorithm would be required to assess and classify the movements and postures.

Machine learning is typically used to solve regression, function approximation, and pattern classification problems, and is particularly well suited to solving large-scale and/or complex problems that are difficult to define mathematically. In existing studies on the assessment of exercise, researchers have employed conventional machine learning (CML) along with IMUs. For example, Whelan et al. [11] used three IMUs to evaluate single-leg squat performance by using the random forest classifier and achieved a classification accuracy of 77%, sensitivity of 77%, and specificity of 78%. By contrast, O’Reilly et al. [12] evaluated single-leg squat performance by using a back propagation neural network combined with a single IMU located on the lumbar region of a subject and achieved a classification accuracy of 56%, sensitivity of 59%, and specificity of 94%. Although the accuracy of squat classification is proportional to the number of IMUs, the use of multiple IMUs is impractical for a self-coaching system because of the inconvenience of wearing multiple IMUs. Although CML can estimate probability density distributions, any unnecessary information or noise in the data will degrade the quality of the results.

By contrast, deep learning (DL) is a technique in which models are constructed by including multiple neural layers for pattern classification or feature learning [13]. Examples of DL models include a variety of structures, such as auto-encoders, restricted Boltzmann machines, convolutional neural networks (CNNs), and recurrent neural networks (RNNs). Among these, CNNs and RNNs are widely utilized in speech recognition and image-processing applications that involve complex calculations. An advantage of DL models is their ability to calculate the weight of input data through multiple layers even if the input data are raw and do not require feature extraction. Recently, many studies have suggested that DL could improve classification performance. If DL with a single IMU can provide sufficient accuracy, it can be used in daily life, considering the convenience it provides. Researchers have shown that multilayer DL architectures can be used to classify exercises, including complex movement patterns, from raw data without manual intervention [14]. Ordóñez et al. [15] used an IMU along with a CNN and an RNN combined with long short-term memory models (RNN–LSTM) to classify various human activities such as opening doors or drinking water. Hammerla et al. [16] used a CNN and an RNN–LSTM to predict the gait stiffness of Parkinson’s patients. Hu et al. [17] used a single IMU to detect surface and age-related differences in walking by using RNN–LSTM.

There are several wearable sensors in the market for fitness in gyms and homes. Most of them measure and recognize the human movement and postures for one part of the body where the sensor is attached. To be used in self-fitness in home training, the smallest number of sensors as possible should be used for convenience and a special algorithm is required to classify the right posture from various aberrant postures from whole body [18]. Many muscle training exercises consist of the repetition of a movement while maintaining a specific posture of the whole body. Hence, most of the current products are not suitable for assessing the exercise posture of the whole body.

The objective of this study is to demonstrate that DL improves the squat posture classification performance obtained from IMU data and to determine the optimal placement of IMUs for self-fitness

## 2. Materials and Methods

### 2.1. Measurement Settings and Experimental Protocol

**Figure 1:**
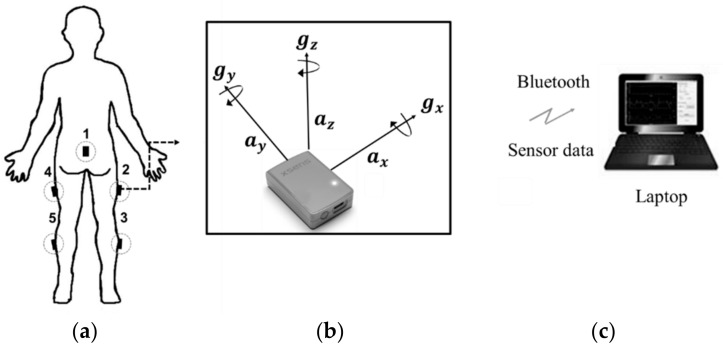
(**a**) Inertial measurement unit (IMU) placement: (1) lumbar region, (2) right thigh, (3) right calf, (4) left thigh, and (5) left calf; (**b**) definitions of axes used by IMUs; and (**c**) laptop used for data processing.

The IMU used in this study was the MTw Awinda (Xsens Inc., Enschede, The Netherlands), which includes multiple motion trackers for real-time 3-D kinematic applications and can measure orientation correctly through a simple setting [19]. In terms of processing, data from a three-axis accelerometer (±2 g) and a gyroscope (±500 o/s) were first collected at 1 kHz and then transmitted via Bluetooth to the computer. In the experiments, the IMUs were placed at the numbered locations shown in Figure 1a, namely the right thigh, right calf, left thigh, left calf, and lumbar region [6]. To maintain the placement location consistency among different participants, the IMUs were attached as follows. As shown in Figure 1a, the IMU positions of number 2 and 4 were located at the one-third points between the patella and pelvis bone. The positions of number 3 and 5 were located at the 1/2 points between the patella and ankle bone. The position of number 1 was located at lumbar spine number 3.

An acceptable squat (ACC) and five forms of aberrant squats that are incorrect squat postures typically adopted by beginners were performed (Table 1). These five forms are associated with the anterior knee (AK), knee valgus (KVG), knee varus (KVR), half squat (HS), and bent over position (BO). The criteria for all of these forms, except HS, were defined by the National Strength and Conditioning Association. The HS form was added because it is frequently observed among incorrect squat postures.

All the participants were trained by a fitness expert to execute the six forms of squats before the experiment; they then performed the squats in random order. The fitness expert corrected the participants’ squat postures to induce the six forms of squats during the experiment, and each repetition duration was 3 s. The participants were allowed to rest for a minute between trials for a particular form of squat, and each trial comprised six repetitions. The participants were 39 healthy people with no prior history of spinal or joint injuries nor diseases. These comprised 20 men and 19 women with an average age of 22.0 ± 2.64 years, an average height of 166.4 ± 7.76 cm, and an average weight of 59.8 ± 9.90 kg. The complete dataset consisted of data from 1404 repetitions performed by the 39 participants. All participants completed the informed consent process before participation. The study procedure was reviewed and approved by the University of Ulsan Institutional Review Board (No. 2018R0002-002).

### 2.2. Preprocessing

**Figure 2:**
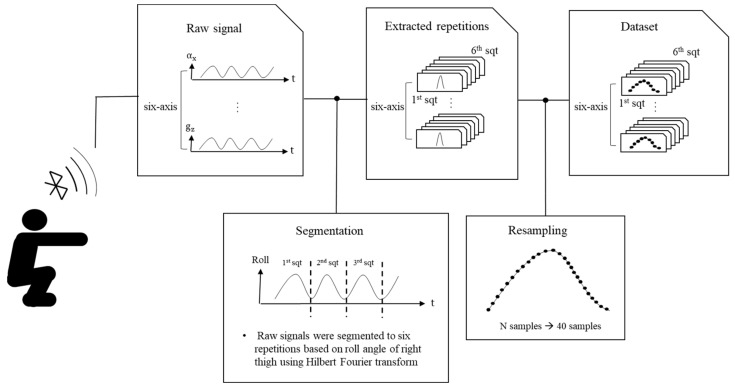
Method of constructing dataset for one trial.

In classifying squat postures, it was essential for each repetition to be extracted by the same number of samples. Because six repetitions were performed in a trial for inducing repetitive exercise and to ensure convenient data collection, each repetition needed to be extracted from a trial with six repetitions. In addition, the start and end timing of one repetition had to coincide with those of the squat data extracted using the other IMUs, which were recording data simultaneously. The extraction of each repetition was achieved using the Hilbert transform based on the roll angle of the right thigh because this angle was observed as the most variable standard with the unaided eye when a pilot study was undertaken. All of the extracted repetitions were raw signals, as shown in Figure 2, and resampled to 40 samples. Figure 2 depicts the method of building the dataset for one trial. The total number of repetitions for all the participants was 1404 for one IMU (39 participants × 6 forms of squats × 6 repetitions).

### 2.3. Classification Algorithms

**Figure 3:**
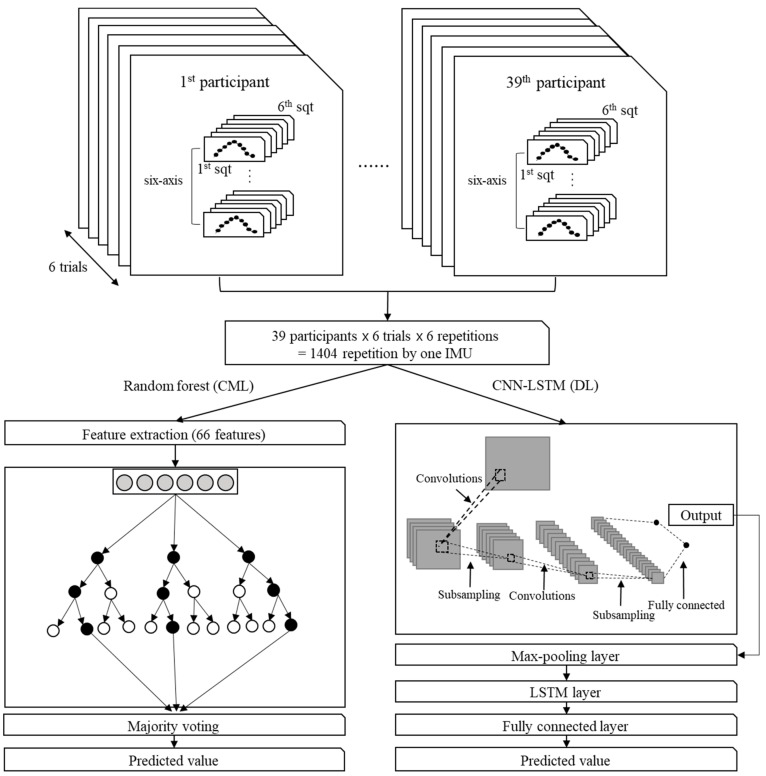
Processes used to train classification models (random forest and convolutional neural network–long short-term memory (CNN–LSTM)) via segmented repetitions of squats.

Different approaches were used to train the classification models with the segmented repetitions of squats in Figure 3. The CML approach was employed for the random forest classification model in this study. This model was originally designed to overcome the disadvantages of decision trees via the bootstrap aggregating (bagging) technique. This model includes the following features: mean, median, max, min, standard deviation, root mean square, range, 25th percentile, 75th percentile, skewness, and kurtosis. The combination of six axes in the IMU and eleven features in the model implied that 66 features were used for classification in this approach.

We used the CNN–LSTM model for the DL approach, which has produced a state-of-the-art performance in recognition of human activity using wearable sensor data [15,20]. As shown in Figure 3, the model comprises three convolutional layers: a recurrent, dense, and softmax layer. The input of the model is the raw data of the size of 6 × 40 for the single IMU and 12 × 40 for the combinations using two IMUs. For the output, the model takes a softmax layer, which generates the probability distribution over the prediction of squat postures. Each convolutional layer had 3 × 3 × N kernels with stride 1, where N doubled each layer from 8 to 32 and used the rectified linear units (ReLUs) as the activation functions. Max-pooling was implemented at the end of every convolutional layer. The recurrent layer employed the long short-term memory (LSTM) units with 64 cells. We implemented the drop-out on each convolutional and dense layer. The model was trained in TensorFlow with the Adam optimizer for 500 iterations [20]. The learning rate was 0.001. Cross-entropy was employed for the loss function. For the sake of regularization, three same models were trained with a different order of inputs and different random weight initializations. The prediction of squat postures was made after averaging the probability distributions produced by these models.

## 3. Results

The data obtained from the 39 participants were divided equally into ten groups, one group of which was randomly selected as the test data while the rest were used as training data before the training model. The training data were used for model training from ten-fold cross-validation. The test data were used as input data in the classification model that had been trained using the other nine groups. This process was repeated 9 more times with changing of the participants for the test group, and the results were averaged. The performance of the classification results was then assessed based on the averaged accuracy, sensitivity, and specificity.

The accuracy of squat posture classification decreased as the number of IMUs reduced (Table 2). For five IMUs, the classification accuracy of CML was 75.4%, and that of DL was 91.7%. In the case of combinations using two IMUs, the combination with IMUs on the right thigh and right calf exhibited the highest performance. In this case, the accuracy was 73.9% for CML and 88.7% for DL. When the IMU on the lumbar region was included, the squat classification accuracy reduced to 34.6% for CML and 57.3% for DL. These results indicate that tracking the IMU displacement on the lumbar region does not aid squat classification. In the case of a single IMU, the best result was obtained from the IMU on the right thigh, with an accuracy of 58.7% for CML and 80.9% for DL.

## 4. Discussion

The experimental results indicated that the squat classification for both single and multiple IMU configurations was more accurate when using DL than when using CML. When five IMUs were used, the classification accuracy of DL was 16.3% higher than that of CML. For a single IMU, the classification accuracy obtained using an IMU on the right thigh for DL was 22.2% higher than that for CML and the classification accuracy obtained using an IMU on the right calf for DL was 18.5% higher than that for CML. Furthermore, although the classification accuracy of 80.9% for 6 classes with a single IMU is insufficient for use in daily life, it is much higher than that in previous studies. This indicates that DL can overcome the limitation of inconvenience from multiple IMUs.

The confusion matrix shown in Table 3 indicates that the DL model trained using data from the right thigh is considered to include some features of the movement of the upper body. Table 3c,d indicates that the accuracy of CML is low for some forms of squats. When the IMU is placed on the right thigh, the squat classification accuracy for BO and AK is lower than that for the other squats. In fact, BO is largely reflected in the movement of the upper body, and AK is reflected in the movement below the knee. By contrast, Table 3a indicates that the classification performance of DL improves the overall accuracy considerably for BO and AK. This is because DL is more powerful than CML in learning the complex mechanisms of closed kinetic chain exercises such as the squat [11,21]. Since each body part moves interactively in the closed kinetic chain exercise, the data from each body part contains hidden features related to the movement of another body part in different squat posture [22,23]. The main challenge of this study was to train these hidden features by optimizing the location of the sensor. We found that the right thigh is the optimal location of the placement of a single IMU.

The current study has several limitations. The dataset used in this study was obtained from 39 participants performing six trials of six forms of squats. However, the size of the dataset was insufficient to optimize the performance of the DL model. In addition, when the squats were classified into acceptable and aberrant squats, the number of acceptable repetitions (6) and aberrant repetitions (30) was unbalanced. Another limitation is that the subjects performed the squats in various postures with the supervision of experts in order to perform squats in specific forms. To develop self-fitness applications, additional research of exercise assessment tools and methods would be required. Reilly et al. [24] developed a mobile app, which automated the process of creating individualized exercise feedback systems. They employed personal classification with a random forest classifier which is specialized in the evaluation of a particular person performing the exercises and requires a smaller dataset. Their systems achieved 89.50% accuracy for assessing aberrant and acceptable squats with a single IMU. By contrast, the deep learning approach of this study can assess six forms of squats at 80% accuracy without an exercise expert with a single IMU. These results can help to develop an application that a beginner can use to get feedback, without coaching, when they perform aberrant squats. With additional work to enhance the performance, our solution could provide meaningful feedback to the persons who exercise at home. It will be helpful to increase the effect of exercise and to prevent the risk of injuries for beginners.

In the future, we plan to investigate whether the squat posture classification can be improved by collecting more data, which would provide better self-fitness results using a single IMU system, and we plan to combine classical motion analysis and deep learning, which could enhance the algorithm’s performance. Another future research area is to identify other DL models that can be used to classify exercises, which would allow the single IMU system to be used for the rehabilitation of athletes and musculoskeletal patients as well. In addition, if the IMU data for other exercises, such as deadlifts, were to be collected, it would allow a single system to be used to classify multiple exercises and provide posture-related instructions to prevent injuries. Finally, we plan to do experimentation that compares the performance of squats on beginners trained by an expert, instructed by a self-fitness application, and a untrained group. 

The single wearable IMU sensor and DL-based posture classification algorithm can be used for a self-training system with a smartphone app at home. In the case of the squat, the sensor attachment on the thigh is convenient and effective for providing feedback on aberrant postures which happens frequently in the beginners. This sensor system can also collect more data from many persons with consent, which can be used to enhance the performance of the algorithm. For the other popular exercises for muscle training, like the crunch, plank, and leg-raise, the concept of this study can be used and integrated as a home trainer with a smartphone and wearable sensor. 

## 5. Conclusions

This study is to demonstrate that deep learning improves the squat posture classification performance from IMU data and to determine the optimal placement of IMUs for self-fitness application. The classification performance of six forms of squat postures using a single IMU on the right thigh showed an accuracy of 80.9% with the deep learning approach. This technology can be used for providing feedback on aberrant squat postures.

## Figures and Tables

**Table 1:** Squat classification showing one acceptable and five aberrant forms.

Squat	Description	Figure	Squat	Description	Figure
Acceptable (ACC)	Normal squat	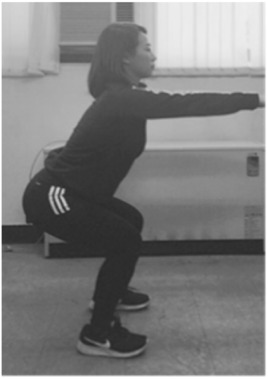	Knee varus (KVR)	Both knees pointing outside during exercise	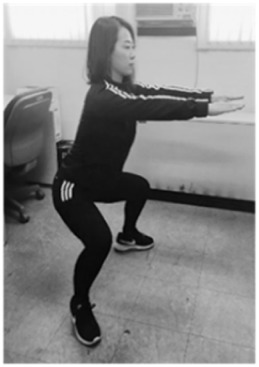
Anterior knee (AK)	Knees ahead of toes during exercise	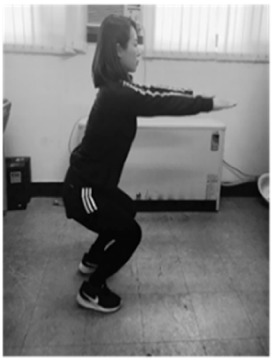	Half squat (HS)	Insufficient squatting depth	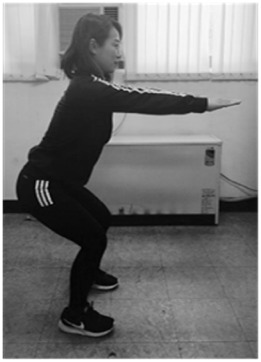
Knee valgus (KVG)	Both knees pointing inside during exercise	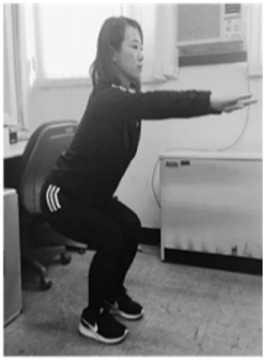	Bent over (BO)	Excessive flexing of hip and torso	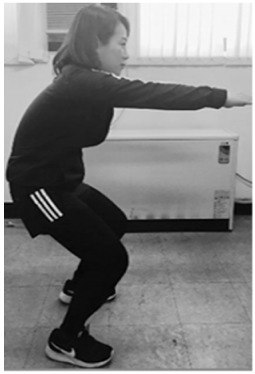

**Table 2:** Squat classification performance of conventional machine learning (CML) and deep learning (DL) for five IMUs, two IMUs, and one IMU.

Number of IMUs	Placement of IMUs	Random Forest (CML)			CNN–LSTM (DL)		
		Accuracy	Sensitivity	Specificity	Accuracy	Sensitivity	Specificity
5 IMUs	Right thigh, right calf, left thigh, left calf, and lumbar region	75.4%	78.6%	90.3%	91.7%	90.9%	94.6%
2 IMUs	Right thigh and lumbar region	63.2%	64.6%	87.6%	83.9%	85.6%	90.4%
	Right thigh and right calf	73.9%	76.8%	89.5%	88.7%	90.5%	95.7%
	Right calf and lumbar region	66.0%	70.1%	86.1%	86.2%	87.1%	87.6%
1 IMUs	Right thigh	58.7%	66.7%	88.9%	80.9%	80.0%	93.1%
	Right calf	57.6%	62.7%	82.2%	76.1%	78.9%	92.8%
	Lumbar region	34.6%	38.6%	68.1%	46.1%	50.3%	79.0%

**Table 3:** Confusion matrix for (**a**) right thigh and (**b**) lumbar region when squats were classified using a single IMU with DL, and confusion matrix for (**c**) right thigh and (**d**) lumbar region when squats were classified using a single IMU with CML. The predicted class refers to the classification provided by an expert, whereas the actual class refers to the classification provided by the mean values of the class in which the subject actually operates.

(**a**) Right thigh with DL								(**b**) Lumbar region with DL							
		Predicted Values								Predicted Values					
		ACC	AK	KVG	KVR	HS	BO			ACC	AK	KVG	KVR	HS	BO
Actual Values	ACC	114	29	43	18	0	30	Actual Values	ACC	80	21	59	49	13	12
	AK	43	92	14	22	20	43		AK	28	82	11	51	44	18
	KVG	24	23	170	0	0	17		KVG	76	11	105	24	16	2
	KVR	28	19	3	168	2	14		KVR	39	33	22	87	40	13
	HS	0	13	0	4	188	29		HS	6	32	13	19	149	15
	BO	34	46	26	4	34	90		BO	15	31	4	17	23	144
(**c**) Right thigh with CML								(**d**) Lumbar region with CML							
		Predicted Values								Predicted Values					
		ACC	AK	KVG	KVR	HS	BO			ACC	AK	KVG	KVR	HS	BO
Actual Values	ACC	114	29	43	18	0	30	Actual Values	ACC	71	22	62	34	28	17
	AK	43	92	14	22	20	43		AK	33	56	18	46	31	50
	KVG	24	23	170	0	0	17		KVG	62	18	87	17	21	29
	KVR	28	19	3	168	2	14		KVR	41	45	38	52	43	15
	HS	0	13	0	4	188	29		HS	23	31	19	49	74	38
	BO	34	46	26	4	34	90		BO	15	31	20	8	16	144
